# Bis(trimethyl­phenyl­ammonium) tetra­bromidocuprate(II)

**DOI:** 10.1107/S160053681000022X

**Published:** 2010-01-16

**Authors:** Kong Mun Lo, Seik Weng Ng

**Affiliations:** a Department of Chemistry, University of Malaya, 50603 Kuala Lumpur, Malaysia

## Abstract

The crystal structure of the title compound, (C_9_H_14_N)_2_[CuBr_4_], consists of two quarternary ammonium cations and a tetra­hedral cuprate anions. Weak C—H⋯Br hydrogen bonding is present between the cation and anion in the crystal structure.

## Related literature

For bis­(4-dimethyl­amino­pyridinium) tetra­bromidocuprate, see: Lo & Ng (2009 ▶).
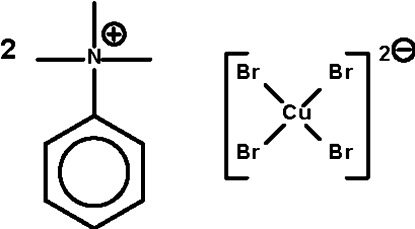


## Experimental

### 

#### Crystal data

(C_9_H_14_N)_2_[CuBr_4_]
*M* *_r_* = 655.60
Monoclinic, 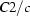

*a* = 16.0146 (11) Å
*b* = 9.8007 (7) Å
*c* = 31.363 (2) Å
β = 94.459 (1)°
*V* = 4907.7 (6) Å^3^
*Z* = 8
Mo *K*α radiation
μ = 7.41 mm^−1^
*T* = 295 K
0.30 × 0.20 × 0.10 mm

#### Data collection

Bruker SMART APEX diffractometer
Absorption correction: multi-scan (*SADABS*; Sheldrick, 1996 ▶) *T*_min_ = 0.215, *T*_max_ = 0.525
25029 measured reflections
4318 independent reflections
2994 reflections with *I* > 2σ(*I*)
*R*_int_ = 0.060

#### Refinement

*R*[*F* ^2^ > 2σ(*F* ^2^)] = 0.048
*wR*(*F* ^2^) = 0.127
*S* = 1.28
4318 reflections
202 parameters
27 restraints
H-atom parameters constrained
Δρ_max_ = 0.61 e Å^−3^
Δρ_min_ = −0.80 e Å^−3^

### 

Data collection: *APEX2* (Bruker, 2008 ▶); cell refinement: *SAINT* (Bruker, 2008 ▶); data reduction: *SAINT*; program(s) used to solve structure: *SHELXS97* (Sheldrick, 2008 ▶); program(s) used to refine structure: *SHELXL97* (Sheldrick, 2008 ▶); molecular graphics: *X-SEED* (Barbour, 2001 ▶); software used to prepare material for publication: *publCIF* (Westrip, 2010 ▶).

## Supplementary Material

Crystal structure: contains datablocks global, I. DOI: 10.1107/S160053681000022X/xu2711sup1.cif

Structure factors: contains datablocks I. DOI: 10.1107/S160053681000022X/xu2711Isup2.hkl

Additional supplementary materials:  crystallographic information; 3D view; checkCIF report

## Figures and Tables

**Table 1:** Selected bond lengths (Å)

Br1—Cu1	2.4055 (11)
Br2—Cu1	2.4057 (11)
Br3—Cu1	2.4136 (11)
Br4—Cu1	2.4039 (11)

**Table 2:** Hydrogen-bond geometry (Å, °)

*D*—H⋯*A*	*D*—H	H⋯*A*	*D*⋯*A*	*D*—H⋯*A*
C2—H2*B*⋯Br3^i^	0.96	2.91	3.840 (9)	164

Symmetry code: (i) 
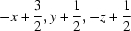
.

**Fig. 1.**
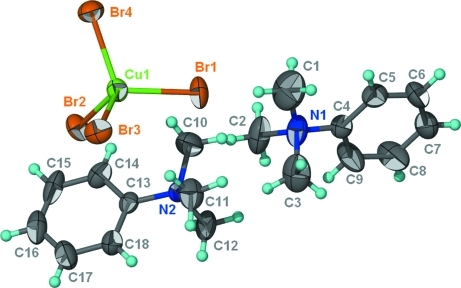
Thermal ellipsoid plot (Barbour, 2001) of bis(trimethylphenylammonium) tetrabromidocuprate at the 50% probability level; hydrogen atoms are drawn as spheres of arbitrary radius.

## supplementary crystallographic information

### Experimental

Copper sulfate pentahydrate (0.52 g, 2 mmol) and trimethylphenylammonium tribromide (0.78 g, 2 mmol) were heated in ethanol (50 ml) for 2 h. After filtering of the reaction mixture, light blue crystals were obtained upon slow evaporation of the greenish-blue filtrate.

### Refinement

Carbon-bound H-atoms were placed in calculated positions (C–H 0.93–0.96 Å) and were included in the refinement in the riding model approximation, with *U*(H) set to 1.2–1.5*U*(C).

The phenyl rings were refined as rigid hexagons of 1.39 ° sides. One trimethylamino group shows somewhat large temperature factors. For investigate possible disorder, all carbon-nitrogen distances were restrained to within 0.01 Å of each other, as were the carbon–carbon distances. The six carbon atoms were restrained to lie within a circle. The temperature factors of the primed atoms were set to those of the unprimed ones. However, this disorder model had short H···H contacts, and the refinement was abandoned. The group was refined without disorder but subject to the same distance restraints. Also, the anisotropic temperature factors were restrained to be nearly isotropic.

The suggested weighting scheme included a large second parameter. This was arbitrarily set at 5.00; this gave a satisfactory Goodness-of-Fit.

### Crystal data

**Table:**

(C_9_H_14_N)_2_[CuBr_4_]	*F*(000) = 2552
*M**_r_* = 655.60	*D*_x_ = 1.775 Mg m^−^^3^
Monoclinic, *C*2/*c*	Mo *K*α radiation, λ = 0.71073 Å
Hall symbol: -C 2yc	Cell parameters from 4958 reflections
*a* = 16.0146 (11) Å	θ = 2.4–22.3°
*b* = 9.8007 (7) Å	µ = 7.41 mm^−^^1^
*c* = 31.363 (2) Å	*T* = 295 K
β = 94.459 (1)°	Prism, blue
*V* = 4907.7 (6) Å^3^	0.30 × 0.20 × 0.10 mm
*Z* = 8	

### Data collection

**Table:**

Bruker SMART APEX diffractometer	4318 independent reflections
Radiation source: fine-focus sealed tube	2994 reflections with *I* > 2σ(*I*)
graphite	*R*_int_ = 0.060
ω scans	θ_max_ = 25.0°, θ_min_ = 1.3°
Absorption correction: multi-scan (*SADABS*; Sheldrick, 1996)	*h* = −18→18
*T*_min_ = 0.215, *T*_max_ = 0.525	*k* = −11→11
25029 measured reflections	*l* = −37→37

### Refinement

**Table:**

Refinement on *F*^2^	Primary atom site location: structure-invariant direct methods
Least-squares matrix: full	Secondary atom site location: difference Fourier map
*R*[*F*^2^ > 2σ(*F*^2^)] = 0.048	Hydrogen site location: inferred from neighbouring sites
*wR*(*F*^2^) = 0.127	H-atom parameters constrained
*S* = 1.28	*w* = 1/[σ^2^(*F*_o_^2^) + (0.05*P*)^2^ + 5.00] where *P* = (*F*_o_^2^ + 2*F*_c_^2^)/3
4318 reflections	(Δ/σ)_max_ = 0.001
202 parameters	Δρ_max_ = 0.61 e Å^−^^3^
27 restraints	Δρ_min_ = −0.80 e Å^−^^3^

### Special details

**Table:**

Geometry. All e.s.d.'s (except the e.s.d. in the dihedral angle between two l.s. planes) are estimated using the full covariance matrix. The cell e.s.d.'s are taken into account individually in the estimation of e.s.d.'s in distances, angles and torsion angles; correlations between e.s.d.'s in cell parameters are only used when they are defined by crystal symmetry. An approximate (isotropic) treatment of cell e.s.d.'s is used for estimating e.s.d.'s involving l.s. planes.

### Fractional atomic coordinates and isotropic or equivalent isotropic displacement parameters (Å^2^)

**Table:**

	*x*	*y*	*z*	*U*_iso_*/*U*_eq_	
Br1	0.55154 (4)	0.30390 (9)	0.16560 (3)	0.0741 (3)	
Br2	0.79401 (4)	0.33759 (8)	0.18242 (3)	0.0617 (2)	
Br3	0.69988 (5)	0.09626 (8)	0.09258 (2)	0.0637 (2)	
Br4	0.67593 (5)	0.49013 (7)	0.07793 (3)	0.0639 (2)	
Cu1	0.67856 (5)	0.30817 (8)	0.12941 (3)	0.0521 (3)	
N1	0.5591 (4)	0.7361 (6)	0.45281 (19)	0.0704 (18)	
N2	0.7051 (3)	0.3098 (5)	0.31014 (15)	0.0434 (13)	
C1	0.5386 (8)	0.7895 (12)	0.4099 (3)	0.167 (5)	
H1A	0.5269	0.8853	0.4115	0.250*	
H1B	0.4902	0.7429	0.3971	0.250*	
H1C	0.5851	0.7753	0.3929	0.250*	
C2	0.6406 (5)	0.7921 (9)	0.4693 (3)	0.114 (4)	
H2A	0.6380	0.8900	0.4691	0.171*	
H2B	0.6832	0.7621	0.4516	0.171*	
H2C	0.6536	0.7608	0.4981	0.171*	
C3	0.5684 (6)	0.5866 (9)	0.4500 (3)	0.123 (4)	
H3A	0.5413	0.5548	0.4235	0.184*	
H3B	0.5432	0.5443	0.4734	0.184*	
H3C	0.6268	0.5636	0.4513	0.184*	
C4	0.4924 (3)	0.7708 (5)	0.47999 (14)	0.0524 (17)	
C5	0.4129 (3)	0.8104 (5)	0.46368 (13)	0.062 (2)	
H5	0.4014	0.8206	0.4343	0.075*	
C6	0.3506 (2)	0.8347 (5)	0.49123 (19)	0.080 (3)	
H6	0.2974	0.8612	0.4803	0.096*	
C7	0.3679 (3)	0.8195 (6)	0.53509 (18)	0.082 (3)	
H7	0.3262	0.8357	0.5535	0.098*	
C8	0.4474 (4)	0.7799 (7)	0.55141 (12)	0.106 (4)	
H8	0.4589	0.7697	0.5808	0.127*	
C9	0.5097 (3)	0.7556 (6)	0.52385 (15)	0.089 (3)	
H9	0.5629	0.7291	0.5348	0.107*	
C10	0.6678 (5)	0.4474 (7)	0.3025 (3)	0.087 (3)	
H10A	0.6929	0.5104	0.3232	0.130*	
H10B	0.6085	0.4431	0.3052	0.130*	
H10C	0.6778	0.4776	0.2743	0.130*	
C11	0.6610 (5)	0.2155 (8)	0.2782 (2)	0.072 (2)	
H11A	0.6783	0.2351	0.2502	0.108*	
H11B	0.6016	0.2282	0.2783	0.108*	
H11C	0.6749	0.1227	0.2856	0.108*	
C12	0.6895 (5)	0.2659 (9)	0.3546 (2)	0.075 (2)	
H12A	0.7211	0.3226	0.3749	0.112*	
H12B	0.7064	0.1726	0.3587	0.112*	
H12C	0.6309	0.2744	0.3586	0.112*	
C13	0.79634 (19)	0.3098 (4)	0.30638 (14)	0.0436 (15)	
C14	0.8423 (3)	0.4295 (4)	0.30389 (15)	0.0606 (19)	
H14	0.8154	0.5135	0.3043	0.073*	
C15	0.9283 (3)	0.4235 (5)	0.30075 (16)	0.080 (3)	
H15	0.9590	0.5035	0.2991	0.096*	
C16	0.9684 (2)	0.2978 (7)	0.30009 (17)	0.083 (3)	
H16	1.0259	0.2938	0.2980	0.099*	
C17	0.9225 (3)	0.1781 (5)	0.30258 (18)	0.079 (3)	
H17	0.9493	0.0940	0.3021	0.095*	
C18	0.8364 (3)	0.1841 (4)	0.30573 (16)	0.062 (2)	
H18	0.8057	0.1040	0.3074	0.074*	

### Atomic displacement parameters (Å^2^)

**Table:**

	*U*^11^	*U*^22^	*U*^33^	*U*^12^	*U*^13^	*U*^23^
Br1	0.0404 (4)	0.1076 (7)	0.0762 (6)	−0.0036 (4)	0.0179 (4)	−0.0069 (5)
Br2	0.0425 (4)	0.0797 (5)	0.0608 (5)	0.0046 (4)	−0.0100 (3)	−0.0093 (4)
Br3	0.0839 (6)	0.0540 (5)	0.0539 (5)	0.0020 (4)	0.0094 (4)	−0.0078 (4)
Br4	0.0684 (5)	0.0573 (5)	0.0654 (5)	−0.0038 (4)	0.0005 (4)	0.0146 (4)
Cu1	0.0433 (5)	0.0610 (5)	0.0518 (5)	0.0006 (4)	0.0032 (4)	0.0000 (4)
N1	0.062 (4)	0.095 (5)	0.057 (4)	−0.014 (4)	0.021 (3)	−0.011 (4)
N2	0.044 (3)	0.047 (3)	0.040 (3)	0.000 (2)	0.008 (2)	0.003 (3)
C1	0.167 (9)	0.222 (10)	0.120 (8)	0.019 (8)	0.064 (7)	0.013 (8)
C2	0.089 (6)	0.127 (7)	0.133 (7)	−0.029 (5)	0.049 (6)	−0.037 (6)
C3	0.118 (7)	0.108 (7)	0.148 (8)	−0.010 (6)	0.050 (6)	−0.058 (6)
C4	0.054 (4)	0.054 (4)	0.050 (5)	−0.001 (3)	0.006 (4)	−0.003 (3)
C5	0.062 (5)	0.073 (5)	0.050 (5)	−0.006 (4)	−0.008 (4)	0.007 (4)
C6	0.049 (5)	0.083 (6)	0.109 (8)	0.014 (4)	0.005 (5)	0.010 (5)
C7	0.070 (6)	0.092 (6)	0.088 (7)	0.022 (5)	0.032 (5)	0.009 (5)
C8	0.112 (8)	0.160 (10)	0.047 (5)	0.059 (7)	0.019 (5)	0.017 (6)
C9	0.069 (5)	0.149 (8)	0.049 (5)	0.043 (6)	0.000 (4)	0.002 (5)
C10	0.070 (5)	0.070 (6)	0.120 (8)	0.008 (4)	0.002 (5)	0.021 (5)
C11	0.055 (5)	0.095 (6)	0.065 (5)	−0.013 (4)	−0.004 (4)	−0.014 (5)
C12	0.058 (5)	0.119 (7)	0.050 (5)	−0.003 (5)	0.022 (4)	0.010 (5)
C13	0.044 (4)	0.054 (4)	0.034 (4)	−0.007 (3)	0.004 (3)	0.002 (3)
C14	0.070 (5)	0.056 (5)	0.056 (5)	−0.016 (4)	0.012 (4)	−0.003 (4)
C15	0.066 (6)	0.115 (8)	0.061 (5)	−0.045 (5)	0.016 (4)	0.000 (5)
C16	0.048 (5)	0.144 (9)	0.057 (5)	−0.011 (6)	0.011 (4)	0.004 (6)
C17	0.052 (5)	0.108 (7)	0.079 (6)	0.016 (5)	0.012 (4)	0.004 (5)
C18	0.047 (4)	0.066 (5)	0.073 (5)	−0.006 (4)	0.009 (4)	0.007 (4)

### Geometric parameters (Å, °)

**Table:**

Br1—Cu1	2.4055 (11)	C6—H6	0.9300
Br2—Cu1	2.4057 (11)	C7—C8	1.3900
Br3—Cu1	2.4136 (11)	C7—H7	0.9300
Br4—Cu1	2.4039 (11)	C8—C9	1.3900
N1—C4	1.457 (7)	C8—H8	0.9300
N1—C1	1.457 (8)	C9—H9	0.9300
N1—C2	1.472 (8)	C10—H10A	0.9600
N1—C3	1.476 (8)	C10—H10B	0.9600
N2—C13	1.475 (6)	C10—H10C	0.9600
N2—C10	1.487 (6)	C11—H11A	0.9600
N2—C12	1.498 (6)	C11—H11B	0.9600
N2—C11	1.499 (6)	C11—H11C	0.9600
C1—H1A	0.9600	C12—H12A	0.9600
C1—H1B	0.9600	C12—H12B	0.9600
C1—H1C	0.9600	C12—H12C	0.9600
C2—H2A	0.9600	C13—C14	1.3900
C2—H2B	0.9600	C13—C18	1.3900
C2—H2C	0.9600	C14—C15	1.3900
C3—H3A	0.9600	C14—H14	0.9300
C3—H3B	0.9600	C15—C16	1.3900
C3—H3C	0.9600	C15—H15	0.9300
C4—C5	1.3900	C16—C17	1.3900
C4—C9	1.3900	C16—H16	0.9300
C5—C6	1.3900	C17—C18	1.3900
C5—H5	0.9300	C17—H17	0.9300
C6—C7	1.3900	C18—H18	0.9300
			
Br4—Cu1—Br2	110.35 (4)	C6—C7—C8	120.0
Br4—Cu1—Br1	111.01 (4)	C6—C7—H7	120.0
Br2—Cu1—Br1	107.98 (4)	C8—C7—H7	120.0
Br4—Cu1—Br3	108.21 (4)	C9—C8—C7	120.0
Br2—Cu1—Br3	107.71 (4)	C9—C8—H8	120.0
Br1—Cu1—Br3	111.54 (4)	C7—C8—H8	120.0
C4—N1—C1	109.5 (7)	C8—C9—C4	120.0
C4—N1—C2	112.2 (6)	C8—C9—H9	120.0
C1—N1—C2	108.7 (6)	C4—C9—H9	120.0
C4—N1—C3	110.4 (6)	N2—C10—H10A	109.5
C1—N1—C3	108.6 (6)	N2—C10—H10B	109.5
C2—N1—C3	107.4 (5)	H10A—C10—H10B	109.5
C13—N2—C10	112.1 (5)	N2—C10—H10C	109.5
C13—N2—C12	108.2 (5)	H10A—C10—H10C	109.5
C10—N2—C12	108.4 (6)	H10B—C10—H10C	109.5
C13—N2—C11	111.4 (4)	N2—C11—H11A	109.5
C10—N2—C11	106.8 (6)	N2—C11—H11B	109.5
C12—N2—C11	109.9 (5)	H11A—C11—H11B	109.5
N1—C1—H1A	109.5	N2—C11—H11C	109.5
N1—C1—H1B	109.5	H11A—C11—H11C	109.5
H1A—C1—H1B	109.5	H11B—C11—H11C	109.5
N1—C1—H1C	109.5	N2—C12—H12A	109.5
H1A—C1—H1C	109.5	N2—C12—H12B	109.5
H1B—C1—H1C	109.5	H12A—C12—H12B	109.5
N1—C2—H2A	109.5	N2—C12—H12C	109.5
N1—C2—H2B	109.5	H12A—C12—H12C	109.5
H2A—C2—H2B	109.5	H12B—C12—H12C	109.5
N1—C2—H2C	109.5	C14—C13—C18	120.0
H2A—C2—H2C	109.5	C14—C13—N2	122.4 (3)
H2B—C2—H2C	109.5	C18—C13—N2	117.6 (3)
N1—C3—H3A	109.5	C15—C14—C13	120.0
N1—C3—H3B	109.5	C15—C14—H14	120.0
H3A—C3—H3B	109.5	C13—C14—H14	120.0
N1—C3—H3C	109.5	C14—C15—C16	120.0
H3A—C3—H3C	109.5	C14—C15—H15	120.0
H3B—C3—H3C	109.5	C16—C15—H15	120.0
C5—C4—C9	120.0	C17—C16—C15	120.0
C5—C4—N1	122.8 (4)	C17—C16—H16	120.0
C9—C4—N1	117.1 (4)	C15—C16—H16	120.0
C6—C5—C4	120.0	C16—C17—C18	120.0
C6—C5—H5	120.0	C16—C17—H17	120.0
C4—C5—H5	120.0	C18—C17—H17	120.0
C5—C6—C7	120.0	C17—C18—C13	120.0
C5—C6—H6	120.0	C17—C18—H18	120.0
C7—C6—H6	120.0	C13—C18—H18	120.0
			
C1—N1—C4—C5	17.7 (7)	C10—N2—C13—C14	11.0 (7)
C2—N1—C4—C5	138.5 (5)	C12—N2—C13—C14	−108.5 (5)
C3—N1—C4—C5	−101.8 (6)	C11—N2—C13—C14	130.6 (5)
C1—N1—C4—C9	−165.8 (5)	C10—N2—C13—C18	−169.6 (5)
C2—N1—C4—C9	−45.1 (6)	C12—N2—C13—C18	71.0 (6)
C3—N1—C4—C9	74.7 (6)	C11—N2—C13—C18	−50.0 (6)
C9—C4—C5—C6	0.0	C18—C13—C14—C15	0.0
N1—C4—C5—C6	176.4 (5)	N2—C13—C14—C15	179.5 (4)
C4—C5—C6—C7	0.0	C13—C14—C15—C16	0.0
C5—C6—C7—C8	0.0	C14—C15—C16—C17	0.0
C6—C7—C8—C9	0.0	C15—C16—C17—C18	0.0
C7—C8—C9—C4	0.0	C16—C17—C18—C13	0.0
C5—C4—C9—C8	0.0	C14—C13—C18—C17	0.0
N1—C4—C9—C8	−176.6 (5)	N2—C13—C18—C17	−179.5 (4)

### Hydrogen-bond geometry (Å, °)

**Table:**

*D*—H···*A*	*D*—H	H···*A*	*D*···*A*	*D*—H···*A*
C2—H2B···Br3^i^	0.96	2.91	3.840 (9)	164

Symmetry codes: (i) −*x*+3/2, *y*+1/2, −*z*+1/2.
